# Integron Gene Cassettes: A Repository of Novel Protein Folds with Distinct Interaction Sites

**DOI:** 10.1371/journal.pone.0052934

**Published:** 2013-01-18

**Authors:** Visaahini Sureshan, Chandrika N. Deshpande, Yan Boucher, Jeremy E. Koenig, Midwest Center for Structural Genomics, H. W. Stokes, Stephen J. Harrop, Paul M. G. Curmi, Bridget C. Mabbutt

**Affiliations:** 1 Department of Chemistry and Biomolecular Sciences, Macquarie University, Sydney, New South Wales, Australia; 2 Department of Biological Sciences, University of Alberta, Edmonton, Alberta, Canada; 3 Department of Biochemistry and Molecular Biology, Dalhousie University, Halifax, Nova Scotia, Canada; 4 University of Toronto, Toronto, Ontario, Canada; 5 ithree institute, University of Technology, Sydney, New South Wales, Australia; 6 School of Physics, University of New South Wales, New South Wales, Australia; 7 Centre for Applied Medical Research, St Vincent's Hospital, Sydney, New South Wales, Australia; University of Technology Sydney, Australia

## Abstract

Mobile gene cassettes captured within integron arrays encompass a vast and diverse pool of genetic novelty. In most cases, functional annotation of gene cassettes directly recovered by cassette-PCR is obscured by their characteristically high sequence novelty. This inhibits identification of those specific functions or biological features that might constitute preferential factors for lateral gene transfer via the integron system. A structural genomics approach incorporating x-ray crystallography has been utilised on a selection of cassettes to investigate evolutionary relationships hidden at the sequence level. Gene cassettes were accessed from marine sediments (pristine and contaminated sites), as well as a range of *Vibrio spp*. We present six crystal structures, a remarkably high proportion of our survey of soluble proteins, which were found to possess novel folds. These entirely new structures are diverse, encompassing all-α, α+β and α/β fold classes, and many contain clear binding pocket features for small molecule substrates. The new structures emphasise the large repertoire of protein families encoded within the integron cassette metagenome and which remain to be characterised. Oligomeric association is a notable recurring property common to these new integron-derived proteins. In some cases, the protein–protein contact sites utilised in homomeric assembly could instead form suitable contact points for heterogeneous regulator/activator proteins or domains. Such functional features are ideal for a flexible molecular componentry needed to ensure responsive and adaptive bacterial functions.

## Introduction

Lateral gene transfer (LGT) allows bacteria to acquire new genetic material and respond to fluid environmental pressures, with a degree of evolutionary change not possible through gradual mutation alone [1], [2]. In recent years, integrons have emerged as key players in microbial LGT and are one of the most efficient genetic elements for the capture and expression of foreign genes [3], [4]. Initially discovered in the context of the spread of multi-drug-resistance in human pathogens, integrons are unique in their ability to combine genes from diverse sources in a linear array suitable for co-expression. The defining feature of integrons is a site-specific recombination system that allows genes that are part of mobilizable elements called gene cassettes to be inserted, excised and rearranged [5], [6].

The integrons predominantly responsible for the spread of antibiotic resistance genes are very similar in DNA sequence. The best example of this is the class 1 integron which essentially represents a single element that has become mobilized by incorporation into other mobile elements such as transposons and plasmids [7], [8]. Class 1 integrons have been responsible for the rapid spread of more than one hundred known resistance genes through a diverse range of pathogens [9]. The class 1 integron, however, is just one example of a very broad group of elements that are phylogenetically diverse [10]. Unlike the class 1, most other integron classes are located in the bacterial chromosome, and have gene cassette arrays which characteristically differ to the class 1 integron arrays. The arrays can be very large, in the case of Vibrios comprising hundreds of cassettes [10], [11]. The genes within the cassettes are remarkably diverse, with only a very tiny fraction encoding identifiable resistance genes [12], [13], and ∼80% shown to carry ORFs with either no known homology or homologous to ORFs of unknown function [10].

The potential impact of the integron in shaping bacterial evolution is largely dependent on the extent to which the mobilised gene cassettes replicate functions already resident within their host genome or contribute additional functions which, while not encoding essential proteins, may provide adaptive traits to the host under certain environmental conditions [14], [15]. Most analyses of the cassette gene pool to date have been focused on sequence-based annotation. Given the high degree of novelty, these approaches are unable to enlighten as to whether the recovered genes encode additional representatives of known protein families, or in fact comprise an additional substantial reservoir of unique functions that heightens the value of an integron array in exploring new ecological niches.

One route to discerning between these two options is through the elucidation of the three-dimensional structure of the encoded protein products, which remains strongly conserved, unlike amino acid sequence [16]. If novel cassettes primarily encode sequence-divergent variants of known proteins, this can be verified through shared fold and geometry of active site; however if the overwhelming novelty of the integron is derived from the presence of many new protein families that we have not seen before, then they are likely to possess novel folds as well.

We have chosen to examine protein structures encoded by the cassette metagenome to discern the degree to which the novel gene sequences truly represent proteins of new fold and function. We have focused on integron gene cassettes recovered by cassette-PCR [17] from uncultured bacteria in environmental samples, as well as from strain isolates of *Vibrio cholerae* and the related *Vibrio metecus* (formerly *paracholerae*). Here, we describe six cassette-encoded proteins within our final group of 19 crystal structures, each found to display a novel fold, and indicating the cassette metagenome to be remarkably rich in new protein families. This group of structures directly accessed from integron arrays provides additional diversity to those genetic elements known to have undergone successful integron-mediated lateral transfer. Their molecular features and organisations contribute new currency to recent discussions assessing the degree to which biochemical function and/or protein network capacity determines transferability of genes [18]–[20].

## Materials and Methods

### Ethics statement

Permits were not required for sampling. Small sediment samples (0.5–1 L) were taken on public land, without causing any disruption in the environment.

### Gene cassette source

Gene cassettes from *V. cholerae*, *V. metecus* (formerly *V. paracholerae*) and environmental sites in the Halifax Harbour (Nova Scotia) vicinity are delineated by the prefixes ‘Vch’, ‘Vpc’ and ‘Hfx’, respectively. **Hfx_cass1**, **Hfx_cass2** and **Hfx_cass5** were isolated from sediments of a salt marsh and two distinct raw sewage effluent outfalls, respectively, as described previously [13]. Strains of *V. cholerae* and *V. metecus* were isolated from a brackish coastal pond (Oyster Pond, Falmouth, MA, USA), as follows. Several water samples (1 ml) were spread directly on thiosulfate citrate bile salts (TCBS) agar (selective for *V. cholerae* family [21]) and incubated overnight at 37°C. Isolated colonies of a yellow colour (sucrose positive [22]) were picked and re-streaked on tryptic soy broth media. After another overnight incubation, isolated colonies were picked and re-streaked on TCBS media and again incubated overnight. This procedure was repeated twice to ensure pure cultured isolates, on which cassette-PCR [17] was performed to isolate integron gene cassettes, including **Vch_cass3** and **Vpc_cass2**. The cassette **Vch_cass14** was sourced by cassette-PCR [17] from the Argentinean ‘Arg3’ O139 strain of *V. cholerae* within a previously described library [23].

### Recombinant protein production

Open-reading frames (ORFs) were cloned with N-terminal histidine tags into p15TV-L vectors (designed by J. Guthrie, University Health Network, Toronto), and expressed in BL21-CodonPlus(DE3)-RIPL *E. coli* (Stratagene, La Jolla). Selenomethionine (SeMet)-derivatised protein was prepared via growth in M9-SeMet media (Medicilon, Shanghai) at 37°C and induction with 1 mM IPTG at 20°C for 20 h. Harvested cells were resuspended in buffer A (300 mM NaCl, 5% v/v glycerol, 1 mM tris(2-carboxyethyl)phosphine, 50 mM 4-(2-hydroxyethyl)-1-piperazine ethanesulfonic acid (HEPES) buffer (pH 7.5)) supplemented with 5 mM imidazole, 1 mM benzamidine and 0.5 mM phenyl methyl sulfonyl fluoride, and lysed by sonication. Soluble His-tagged proteins were purified from the supernatant by Ni-nitriloacetic acid affinity chromatography (Qiagen, Mississauga) at 4°C, eluting with batch-wise applications of buffer A containing increasing concentrations of imidazole (5, 30, 250 mM). After addition of ethylenediamine tetraacetic acid (EDTA) to 1 mM, the purified fraction was equilibrated into buffer A (lacking the glycerol component) at 4°C.

**Vch_cass14** was cloned with an N-terminal histidine tag into pET15b vectors (Merck, Kilsyth) and expressed and purified in BL21(DE3) Rosetta *E. coli* (Merck, Kilsyth), as described [24]. SeMet-derivatised protein was produced in 1 l cultures via auto-induction using PASM-5052 media [25]. Purified protein was dialysed into 50 mM Tris buffer pH 9.0, following screening of a range of solubilisation buffers [24].

### X-ray crystallography

Protein samples were concentrated (>10 mg/ml) and placed at room-temperature in sitting-drop format with 1∶1 and 2∶1 ratios of protein and precipitant in total volumes of 0.4–2.0 µl (Intelliplates, Art Robbins; Flexible Microtest Plate, Becton Dickinson) against in-house sparse-matrix crystallisation screens [26]. **Vch_cass14** was additionally trialled with a full range of commercial screens (Qiagen). Diffraction-quality crystals were grown in either hanging- or sitting-drop formats under the following conditions: **Hfx_cass1** - 25% PEG3350, 0.2 M ammonium sulphate, bis-Tris buffer, pH 6.5; **Hfx_cass2** - 20% PEG3350, 0.2 M tri-lithium citrate; **Hfx_cass5** – 29% PEG3350, 0.1 M HEPES buffer pH 7.5; **Vch_cass3** **-** 0.1 M sodium acetate, 2 M sodium formate, pH 4.6; **Vch_cass14 –** 20% PEG3350, 0.2 M lithium acetate; **Vpc_cass2 –** 20% PEG3350, 0.2 M di-sodium tartrate, treated with chymotrypsin [26].

All diffraction data were collected at 100 K at the Advanced Photon Source (Argonne National Laboratory, Illinois). At beamline 19-ID, collection utilised an ADSC QUANTUM 315 CCD detector and 0.979 Å X-rays (**Hfx_cass1**, **Hfx_cass5, Vch_cass3** and **Vpc_cass2**); at beamline 19-BM, a SBC-3 CCD detector and 0.979 Å X-rays were used (**Hfx_cass2**); and at beamline 23-ID-B, a MARMosaic 300 CCD detector and 1.033 Å X-rays were utilised (**Vch_cass14**). Data were processed using MOSFLM [27], SCALA [28], HKL3000 [29], SCALEPACK [30] and CCP4 software [31]. The PHENIX suite [32] was used to solve phases from Se-derivatised methionines within each protein chain and for automated building and refinement. Manual model-building of protein chains, water molecules and bound components was performed with Coot [33]. Topology and parameter files for sulphate and acetate ions in the **Hfx_cass1** and **Vch_cass14** models were obtained from the HIC-Up database [34]. Electron density of the linear molecule observed within the cavity of **Vch_cass14** did not resemble any of the crystallisation components, and has been left unmodelled. Model geometry was assessed with PROCHECK [35] and MOLPROBITY [36].

Statistics for solution and refinement of the structures are presented in Table 1. Structures in this work display 1.80–2.30 Å resolution, with crystallographic and free *R-*factors ranging between 0.173–0.195 and 0.224–0.241, respectively. All six structures have been deposited in the Protein Data Bank (PDB) under the following accession codes: **Hfx_cass1**, 3FUY; **Hfx_cass2**, 3FXH; **Hfx_cass5**, 3IF4; **Vch_cass3**, 3FY6; **Vch_cass14**, 3IMO; and **Vpc_cass2**, 3JRT.

**Table 1:** Crystallographic data collection and refinement statistics for structure determination.

Data set	Space group	a.s.u^a^	Unit cell (Å)	Unit cell (°)	Resolution (Å)	Total Refl.	Unique Refl.	<*I/σ*>	Complete-ness (%)	Multi-plicity	R_merge_ b
Hfx_cass1 (outer shell)	P3_2_	3	a = 71.6 b = 71.6 c = 92.7	α = 90 β = 90 γ = 120	2.00 (2.11–2.00)	202528 (29380)	35948 (5287)	7.4 (0.9)	100.0 (100.0)	5.6 (5.6)	0.082 (0.560)
Hfx_cass2 (outer shell)	C2	1	a = 40.7 b = 66.8 c = 48.9	α = 90 β = 113.5 γ = 90	1.84 (1.87–1.84)	70004 (1789)	10187 (252)	8.8 (3.0)	97.3 (95.8)	6.9 (7.1)	0.087 (0.669)
Hfx_cass5 (outer shell)	P2_1_	4	a = 61.3 b = 44.5 c = 82.5	α = 90 β = 109.8 γ = 90	2.18 (2.24–2.18)	151462 (3052)	21618 (763)	10.3 (2.6)	97.6 (68.6)	7.0 (4.0)	0.093 (0.531)
Vch_cass3 (outer shell)	P2_1_2_1_2_1_	4	a = 50.3 b = 97.4 c = 115.4	α = 90 β = 90 γ = 90	2.10 (2.21–2.10)	129402 (18698)	33584 (4815)	11.4 (1.8)	99.3 (99.2)	3.9 (3.9)	0.088 (0.403)
Vch_cass14 (outer shell)	C2	4	a = 119.9 b = 64.7 c = 81.98	α = 90 β = 131.3 γ = 90	1.80 (1.90–1.80)	164116 (23623)	43702 (6365)	10.6 (1.1)	99.9 (100.0)	3.8 (3.7)	0.066 (0.629)
Vpc_cass2 (outer shell)	P6_4_	1	a = 85.5 b = 85.5 c = 46.7	α = 90 β = 90 γ = 120	2.30 (2.42–2.30)	123960 (18536)	8801 (1281)	15.6 (1.6)	99.9 (100.0)	14.1 (14.5)	0.072 (0.473)

Refinement statistics	Hfx_cass1	Hfx_cass2	Hfx_cass5	Vch_cass3	Vch_cass14	Vpc_cass2					
Water molecules	163	96	132	323	209	31					
R_cryst_/R_free_	0.199/0.234	0.191/0.241	0.183/0.233	0.173/0.224	0.195/0.230	0.193/0.234					
Reflections in R_cryst_/R_free_	35948/6704	9942/1000	21127/1951	33584/2000	41948/4294	8782/871					
Resolution range (Å)	92.85-2.00	50.00-1.84	50.00-2.18	74.43-2.10	45.08-1.80	42.76-2.30					
Overall isotropic B-factor (Å^2^)	38.8	39.7	34.4	28.5	30.6	66.9					
r.m.s.d. bond lengths (Å)	0.007	0.007	0.005	0.006	0.007	0.008					
r.m.s.d. bond angles (°)	0.989	0.924	0.876	0.940	0.944	0.968					
Ramachandran plot^c^:											
Most favoured region (%)	97.0	98.2	96.6	99.4	95.8	95.8					
Additionally allowed (%)	3.0	1.8	2.9	0.6	3.7	3.6					

a Chains per asymmetric unit (a.s.u).

b ∑∑*_i_*|I*_hi_ −* I*_hi_*|/∑∑*_i_*I*_h_*, where I*_h_* is the mean intensity of reflection *h.*

c From MolProbity [36].

Structures were analysed for homologues using facilities within DALI [37], as well as the SSM-server [38] and FATCAT [39]. Sequence homology searches were performed using Blast [40] and TBlastN [40] against the non-redundant and environmental non-redundant databases. Multiple sequence alignments were done with CLUSTALW 2 [41]. Analysis of subunit interactions, surface clefts, and detection of matches to active site, ligand-binding and DNA-binding templates (August 2011) utilised PISA (version 1.18) [42], IsoCleft Finder [43] and ProFunc [44] tools. ProtParam [45] formulae were utilised to calculate M_r_ values. Molecular images were generated with Pymol [46].

### Size-exclusion chromatography

Oligomeric states were determined for some recombinant protein products by size exclusion chromatography performed at 0.5 ml/min on a Superdex 200 column (10×300 mm, GE Healthcare) pre-equilibrated with 50 mM HEPES buffer (pH 7.5, with 300 mM NaCl. For **Vch_cass14**, the running buffer was 50 mM Tris (pH 9.0). Elution volumes were calibrated with size standards (13.7–440 kDa) and blue dextran (GE Healthcare).

## Results

**Figure 1:**
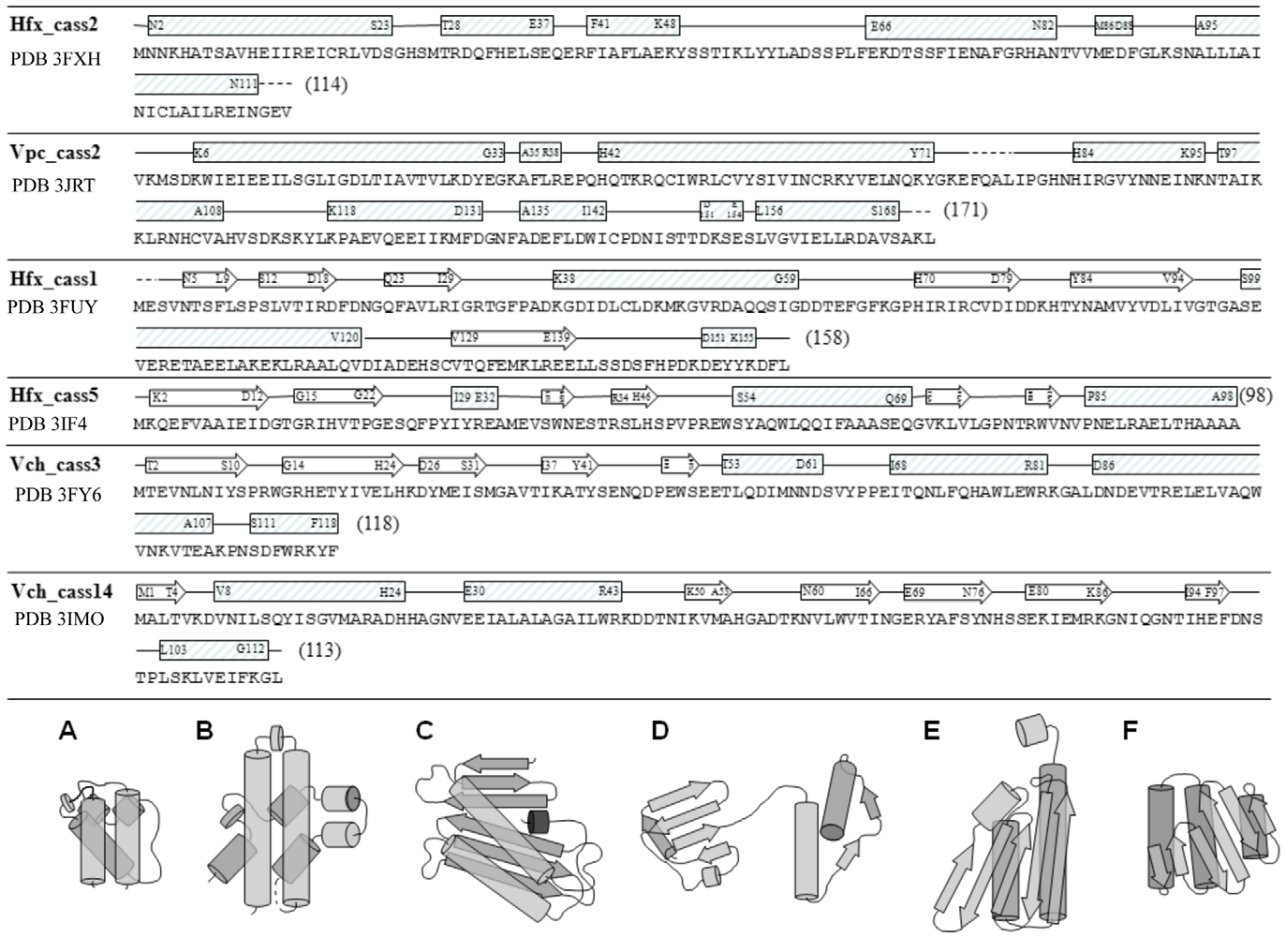
Structural elements and topology of novel gene cassette proteins. Sequences of gene cassette ORFs aligned with secondary structural features observed within crystal structures (arrows, β-strands; blocks, α-helices; dashed lines, undefined flexible regions). Sequences do not include additional affinity tags used for recombinant production. Schematic diagrams show structures of monomer forms for proteins **A. Hfx_cass2, B. Vpc_cass2, C. Hfx_cass1, D. Hfx_cass5, E. Vch_cass3** and **F. Vch_cass14**.

The gene cassettes described in this study arise both from chromosomal integron arrays of multiple *Vibrio* strains (*V. cholerae* and *V. metecus*), as well as metagenomic DNA extracted from environmental sites of varying anthropogenic disturbance [13]. Crystal structures have been solved for **Hfx_cass1** (pristine salt marsh), **Hfx_cass2** (sewage outfall A), **Hfx_cass5** (sewage outfall B), **Vch_cass3** (*V. cholerae*, Oyster Pond isolate), **Vch_cass14** (*V. cholerae*, Arg3 strain) and **Vpc_cass2** (*V. metecus*, Oyster Pond isolate). Amino acid sequences of these structural targets are depicted in Figure 1. The cassettes sampled directly from the environment had no sequence homologues (**Hfx_cass1** and **Hfx_cass2**), or none outside of the cassette metagenome (**Hfx_cass5**). The remaining three *Vibrio*-associated cassettes encoded ORFs displaying some sequence identity (∼40–60%) to hypothetical proteins of no annotated function within various gram-negative bacterial genomes. The three-dimensional structures and topologies of the six proteins described here, encompassing all-α, α/β and α+β fold families, do not directly match previously known structures and reveal new folds not present in current structural databases.

### All-alpha fold structures

#### Hfx_cass2

**Figure 2:**
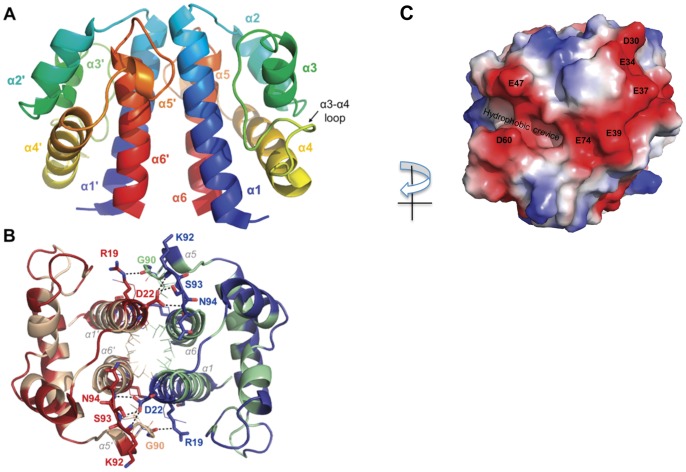
Helical packing in the Hfx_cass2 dimer (PDB 3FXH). **A**. Ribbon depiction with colour spectrum from N-terminus (blue) to C-terminus (red) for each chain. **B.** Dimer interface engages hydrophobic residues from Chain A (tan) and Chain B (green). Acidic groups located either side of a small hydrophobic pocket on the external face are indicated (red). **C.** Electrostatic surface potential of the dimer surface. Key acidic features (Glu47, Asp60 and helix 2 side chains) are labelled.

The crystal structure of Hfx_cass2 (PDB 3FXH) depicts a homodimer incorporating a compact all-α fold of six helical segments. N- and C-terminal helices of each chain lie anti-parallel to one another across a hydrophobic interface (shown in Figure 2), creating a core central bundle of helices (α1, α6, α1′ and α6′). Hydrophobic side chains from helix 1 (Ile14, Leu91) and helix 6 (Leu97, Ile100, Leu104 and Leu107) of both chains bury ∼1800 Å2 surface area to stabilise the dimer.

The externally exposed face of each protomer is, by contrast, markedly acidic. It displays a pair of short helices (α2 and α3) angled at ∼60°, below which helix α4 extends fully. A prominent 17-residue loop connecting helices α3 and α4 also contributes at this site, positioning the Asp60 side chain opposite Glu47 (helix 3) across a hydrophobic triangular-shaped crevice (see Figure 2C). Residues 60–66 of the loop appear to be most flexible, possibly modulating access for any interacting ligand at this position. Running almost perpendicular to the cavity, helix 2 side chains (Asp30, Glu34, Glu37, Glu39) and Glu74 (from helix 4) create an exposed acidic stripe, extending 19 Å. The protein encoded within this single gene cassette thus presents a prominent binding groove, potentially gated by acidic residues.

No structural or sequence-based homologues are currently identifiable for this novel variant of helical fold. Recombinant **Hfx_cass2** preparations are found to organise as stable dimers in solution.

#### Vpc_cass2

**Figure 3:**
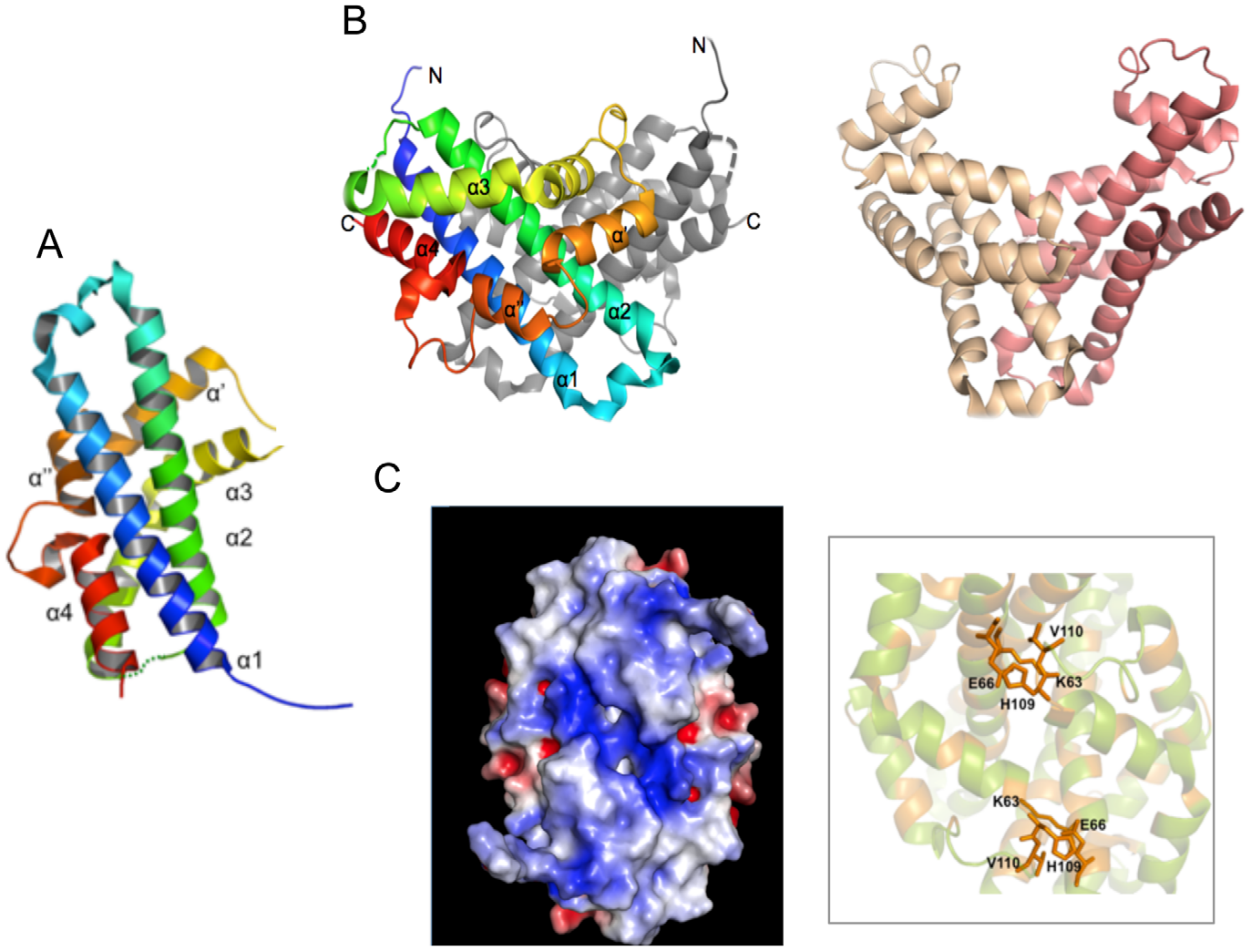
The Vpc_cass2 dimer (PDB 3JRT). **A.** Ribbon of monomeric unit with colour spectrum (blue to red) across six helical components (α1, α2, α3, α′, α″, α4), named as indicated. A loop of weak density connecting helices 2 and 3 is represented by dotted line. **B.** Contrasting shapes of dimers of **Vpc_cass2** (left panel) and structural relative HI0074 from *Haemophilus influenza* [55] (right panel). **C.** View (left panel) across **Vpc_cass2** dimer interface shown as electrostatic surface (in blue to red from +5 to −5 kbT/e [57]), highlighting the basic cluster unique to this protein. Right panel provides segmental view of dimer ribbon (green) and side chains of putative active site residues of **Vpc_cass2** straddling the dimer interface. Residues conserved in **Vpc_cass2** and its sequence homologs from *Shewanella* and *Moritella sp* are coloured orange.

The structure of **Vpc_cass2** (PDB 3JRT) is based on a four-helix bundle, in this case interrupted by two helical extensions, helices α′ (residues 118–131) and α″ (135–142) wrapping about the bundle at midpoint. The resulting helix order is 1-2-3-α′-α″-4, as depicted in Figure 3. Helix 4 is disrupted by an exposed and markedly flexible loop (Cys143-Leu156) which incorporates a β-turn (145–148).

In the crystal, the **Vpc_cass2** bundle is organised into a relatively globular dimer through intermolecular interactions engaging ∼25% of residues of each chain. Clusters of hydrophobic side chains on the surfaces of helix 1, helix 2 and the loop connecting helices α′and α″ (Val107, Ala108, Val1100) mediate the dimeric interface. These same helix and loop components also contribute to hydrogen bonding and salt bridge stabilisation of the dimer. A spread of basic side groups is a distinctive feature of the exposed surface of the dimer, incorporating Arg62, Lys100, Lys101 and Arg103 sidechains from both chains (Figure 3).

The identification of structural relatives for **Vpc_cass2** is somewhat obscured by its relatively simple helical form, but diverse tools indicate a relationship to the KNTase_C (kanamycin nucleotidyltransferase C-terminal domain) clan (CL0291) of proteins [47]. The fold homology is most readily seen for the 4-helix families specifically annotated as NTase_sub_bind (PF08780: PDB 1JOG, rmsd 2.9 Å; PDB 1WTY, rmsd 3.2 Å) and DUF86 (PF01934: PDB 1YLM, rmsd 3.3 Å). The nucleotidyltransferases of this clan organize as two component systems (independent domains or gene pairs encoding a hetero-oligomeric complex): an α/β domain for nucleotide binding and a separate domain (often helical) providing for a wide range of substrate types. It is the defined helical substrate-binding domains to which **Vpc_cass2** is related.

A comparison of these closest structural relatives with **Vpc_cass2** convincingly shows our new structure to possess distinct features, most obviously (i) a unique loop disrupting helix 4, (ii) elongation of helices α′ and α″, and (iii) the absence of additional helix between helices 2 and 3. Although there is no relationship at the sequence level, the majority of structures defined across this clan consistently show dimeric organisation mediated largely through hydrophobic residues of helix 2. Significantly, the packing geometry of these various dimeric structures are markedly different. Often the pair of helix bundles are angled, so creating a deep “V”-shaped interdomain cleft embellished with distinct basic patches, perhaps suitable for nucleic acid binding. However, in the case of **Vpc_cass2**, the alignment angle between chains is considerably different, resulting in a compact and relatively flat surface (panel B, Figure 3).

Two close sequence homologues of **Vpc_cass2** can be seen within the genomes of *Shewanella baltica* and *Moritella spp*., displaying ∼50% amino acid identity (and which retain 62–70% sequence homology). Spatial mapping of the invariant amino acids onto the **Vpc_cass2** fold reveals strong preservation of the hydrophobic residues forming the dimer interface, suggesting retention of the dimeric structure. An additional cluster of conserved residues projects across the interface in the vicinity of the carboxyl end of helix 2, incorporating Lys63 and Glu66 side chains grouped with His109′ (Figure 3C). This preserved feature likely contributes to the biochemistry of a substrate site for this protein family. The basic surface residues of the **Vpc_cass2** dimer, however, appear to be unique to just this member of the sequence group.

### Alpha/beta fold structure

#### Hfx_cass1

**Figure 4:**
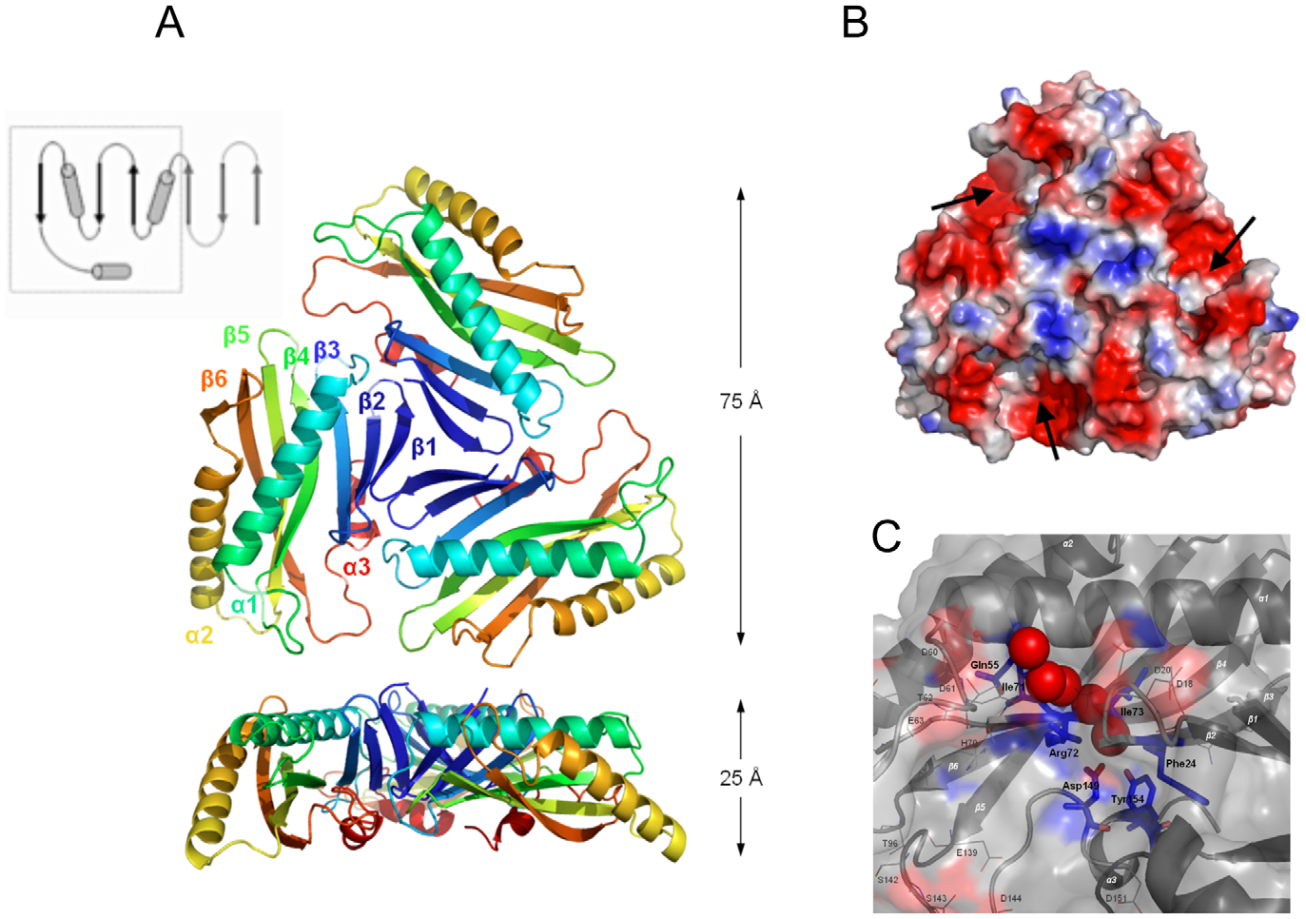
The distinctive flattened shape of trimeric Hfx_cass1 **(PDB 3FUY).** **A.** Dimensions highlight distinctive flattened form. Topology map indicates (dashed line) subfold segment found in zinc transporter domain CzrB [50], to which **Hfx_cass1** is not functionally related. **B.** Electrostatic surface potential of the trimer surface highlights polar cavities (arrowed) and exposed acidic clusters on external loops. **C.** Residues from β-strands 3 and 4 form a polar crevice (blue) surrounded by surface loops containing charged residues (red). Solvent molecules trapped within the crevice are shown (spheres).

**Hfx_cass1** (PDB 3FUY) is a trimer of distinctive flattened shape (75 Å×25 Å), in which each protomer adopts a three-layered α/β-fold. Each subunit contains a mixed six-stranded central sheet underlying two extended α-helices and flanked on the alternate face by a 3_10_-helix (Figure 4). Weaving outwards from the centre of the trimer, strands β1 and β2 of each sheet form a simple meander, followed by two inverted β-α-β motifs. Whilst the β-α-β motif incorporating strands β5/β6 utilises conventional topology, the first motif (connecting β3/β4) involves a rare left-hand cross-over (observed only in ∼1.5% of supersecondary structures [48]). The novel crossover loop is relatively long, incorporating a G/P-rich segment of eight residues (G30-D37).

Within the flattened structure of **Hfx_cass1**, only a small proportion (∼11%) of residues engage in interactions across subunit interfaces. The trimer is primarily stabilised by hydrophobic contacts from β1 strand residues at the centre to neighbouring loop features (β1-β2, β2′-β3′, β3-α4 and β6′-α3′). A salt bridge engages two adjacent loops (His82/Asp144′) and hydrogen bonds occur between residues on the inner β-strands and nearby loops and the C-terminal 3_10_-helix (Ser12′-Asp151-Gly33′).

As a result of a β-bulge between β4 and the adjacent β5 strand (at residues 74, 75 and 89), strands β3 and β4 are splayed apart, interacting at their carboxyl ends only. The β-bulge secondary feature has long been associated with active sites of proteins [49]. In the case of **Hfx_cass1**, the two splayed strands create a narrow polar cavity, bound above and below by helices 1 and 3, and occupied by water molecules in all three subunits (Figure 4). Surrounded by pronounced acidic clusters, largely from side chains on loop features, this region has the appearance of a functional binding site. Acidic side chains contributing to the open cavities adjacent to each cleft include Asp79 and Asp80 (β4′-β5′ loop) as well as Glu139, Asp144, Asp149 (β6-α3 loop).

We note that in the packing of our **Hfx_cass1** crystal, these proposed binding sites engage surface side chains from protomers of neighbouring trimers. A search against a database of cognate binding sites [43], identified some features at this location common to enzymes utilising nucleotide-based cofactors (e.g. adenosine and/or nicotinamide moieties). However, **Hfx_cass1** displays none of the known sequence motifs for binding these cofactors.

While no direct sequence or structural homologues of **Hfx_cass1** have yet been reported, some sub-fold similarity is detected to the zinc transporter CzrB (PDB 3BYP [50]) from *Thermus thermophilus.* The cytosolic zinc-binding domain of CzrB, an integral membrane transporter, aligns (2.8 Å rmsd) with C-terminal residues (38–148) of **Hfx_cass1**. The CzrB fold incorporates a helix followed by a β strand and an inverted β-α-β motif, hence overlapping a portion of the β-α-β repeat motifs of Hfx_cass1. In CzrB, the domain presents a cluster of zinc-binding residues for metal chelation and controls a dimerisation event critical to function [50]. However, these active site residues are not replicated in the equivalent strands (β3-β6) of **Hfx_cass1**, to which there appears to be no functional relationship.

### Alpha + beta fold structures

#### Hfx_cass5

**Figure 5:**
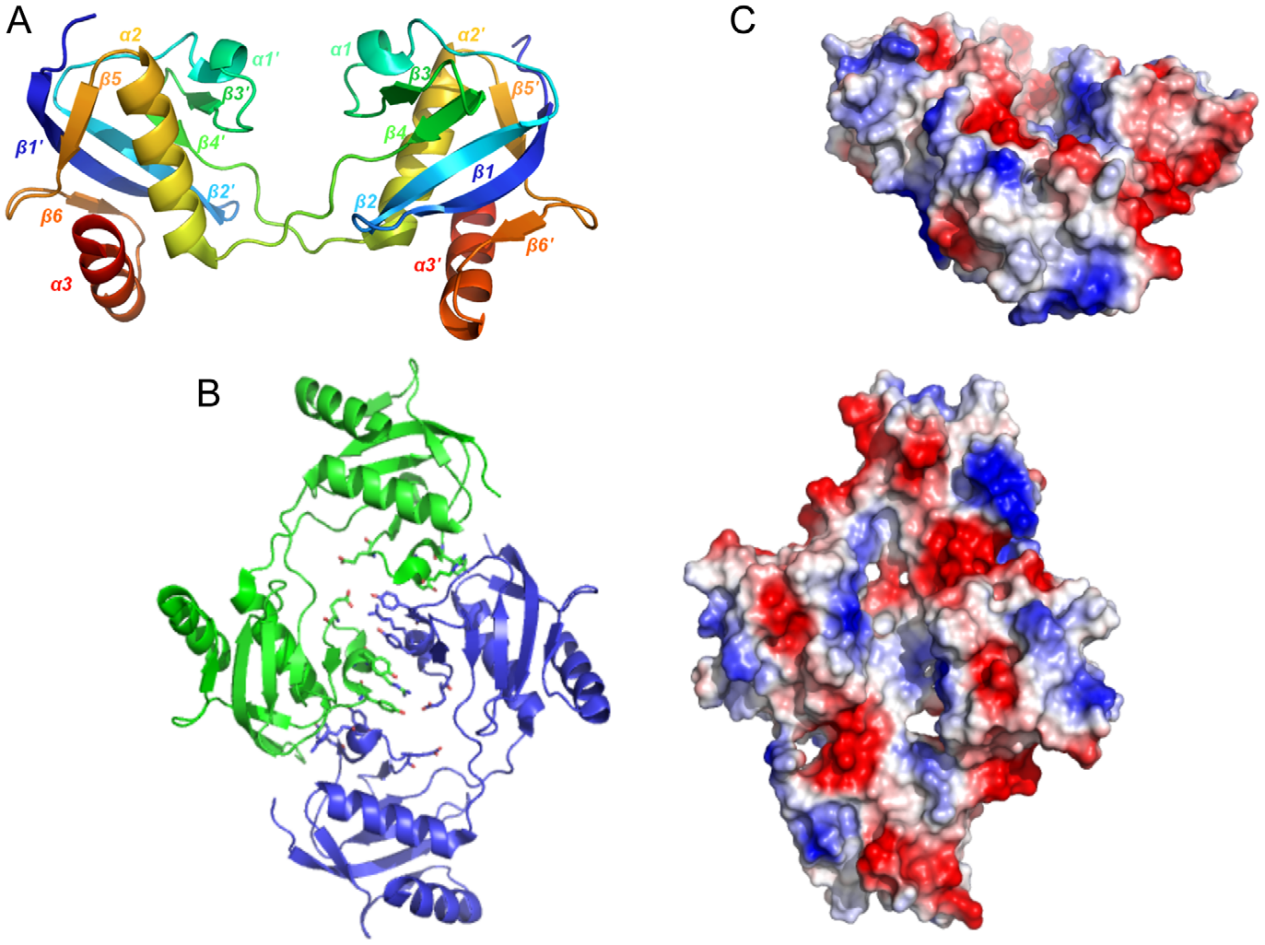
Hfx_cass5 contains domain-swapped dimers (PDB 3IF4). **A.** Ribbon depiction of two domain-swapped chains, each coloured from N-terminus (blue) to C-terminus (red). **B.** Tetrameric organisation depicted in ribbon form (left panel), showing engagement between two dimers (green and blue), each comprising two chains. Side chain stacking of key contacting residues (Tyr28, Tyr30, Arg31 and Glu35) is depicted. Corresponding view of electrostatic surface is also shown (right), with deep cleft centrally positioned. **C.** Rotated view of electrostatic surface, positioned to emphasise narrow dimensions of this basic slot along one tetramer surface.

The 2.18 Å structure of **Hfx_cass5** (PDB 3IF4) reveals a symmetrical domain-swapped dimer of compact α+β domains. As shown in Figure 5, this structure forms half of a structurally asymmetric tetramer. One face of each domain contains a five-stranded β-sheet, predominantly antiparallel in nature (strand order: 6′1243). Overlaying this sheet, creating an alternate face to the domain, are two helices (α2′ and α3′) and a parallel β-ribbon formed by strand β5′ and the N-terminal segment of β1. An extended loop and short 3_10_-helix (α1: Tyr28-Ala33) between strands β2 and β3 connect the two domain faces. Residues 1–46 of each chain contribute strands β1-β4 and helix α1 of one domain; a Pro-containing segment with slightly elevated B-factors creates the inter-domain linker; residues 54–98 form the alpha helices and intervening strands (β5′ and β6′) within the second domain.

At the centre of the crystallised tetramer, the 3_10_ helices at the edges of two opposing subunits come into contact via well-ordered stacking of protruding polar and charged side chains (Tyr28, Tyr30, Arg31, Glu35; see Fig. 5B). This contact means that the 3_10_ helices from the remaining two domains are separated further out along the tetrameric interface.

The flattened nature of the tetramer and the asymmetrical interactions of its component dimers results in two large faces with markedly different surface features. On one face, a narrow slot (Figure 5C) is formed by relatively close juxtaposition of the beta sheets from the two domains linked via contacting 3_10_ helices. This cleft is lined with basic features (side chains Arg16, His46 and Arg51). On the opposite face, the equivalent slot is very wide, due to the diagonal separation of the sheet components about the 3_10_ helix interface.

A small group of sequence homologs to **Hfx_cass5** have been described. Of these, three closest relatives (55–71% identity) are also encoded as gene cassettes. Source environments include the same sewage outfall as **Hfx_cass5**, a geographically distinct sewage outfall in Halifax, Canada, and an industrial site in Australia. Two more remote relatives are seen in species of *Pseudoxanthomonas* (US feedstock culture; 48% identity) and *Shewanella* (Pacific hydrothermal vent; 34% identity). Alignment of this **Hfx_cass5** family of sequences (given in Figure S1) immediately reveals residues comprising the 3_10_-helix (α1) and the inter-domain linker to be highly conserved across the group. Preserved residues include the key charged and aromatic groups mediating tetrameric organisation: Tyr30, Arg31, Glu32, Glu35, Arg51, Glu52. This conservation emphasizes some pressure to maintain a functioning asymmetric tetramer across the **Hfx_cass5** family. The tetrameric association also requires retention of the inter-module linker sequence, preserved as –PxPRE/QW- across the sequence group. This linker segment also contains Arg sidechains conserving the basic chemistry of surface clefts on the two faces of the tetramer.

This new structure of **Hfx_cass5** does not match any classified SCOP fold or previously described sub-fold. Some topological similarity is detected between the N-terminal segment of **Hfx_cass5** and the ββαββ structure motif found in domain II of the bacterial ribosome recycling factor (RRF) family (rmsd 2.9–3.1 Å). Outside of the shared structural motif, however, the **Hfx_cass5** and RRF folds significantly differ, and the pair are not considered to be structural relatives.

#### Vch_cass3

**Figure 6:**
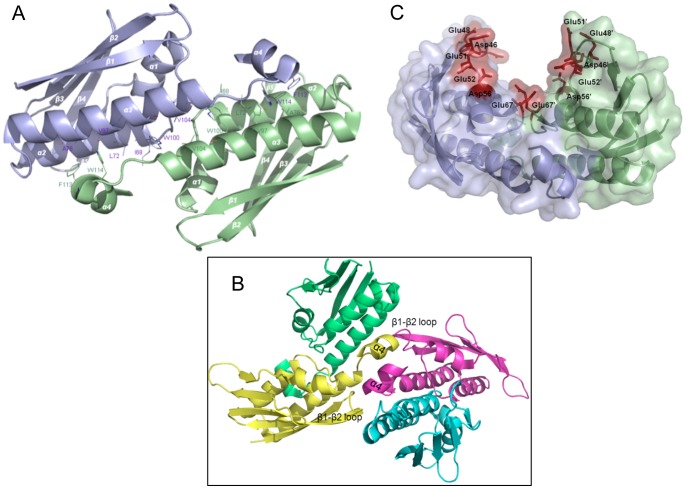
The dimer of Vch_cass3 (PDB 3FY6). **A.** The two chains of the **Vch_cass3** dimer are illustrated, including stabilising hydrophobic residues from helices 2, 3 and 4. **B** View depicting **Vch_cass3** dimers interacting via helix 4 and β1-β2 loop regions. Located in a single asymmetric unit are dimer 1 (yellow, green chains) and dimer 2 (pink, cyan chains). **C.** The proposed functional site formed by the dimer (blue and green chains) is distinct from any conserved regions, and consists of acidic residues (red) belonging to β5, and helices 1 and 2.

The structure of **Vch_cass3** (solved to 2.10 Å, PDB 3FY6) reveals a dimer in which each protomer adopts a two-layered α+β fold. The N-terminal portion (1–61) of each chain forms an anti-parallel β-sheet of five strands (12345 topology) which curves around a pair of antiparallel helices, 2 and 3 (encompassing residues 68–107). The C-terminus of each chain is extended by a short helix α4 (residues 112–118). Within the dimer, helices 2 and 2′ stack essentially end-to-end, creating a distinctive central helical core in conjunction with helices 3 and 3′ (Figure 6). The two opposing sheet features are thus separated across the elongated dimer, both presenting exposed faces to solvent. Each C-terminal helix 4 is angled across the neighbouring set of helices, 2′ and 3′. This helix contains a significant number of aromatic side chains (Phe113, Trp114, Tyr117, Phe118) which contribute hydrophobic and hydrogen-bonding stability to the dimeric interface.

In the crystal structure of **Vch_cass3**, the asymmetric unit depicts association of dimers into a tetrameric structure. The tetrameric association incorporates interactions between helix 4 and the β1′-β2′ loop of adjacent dimers (Figure 6B). Such crystal interactions may be indicative of interactions possible for heterogenous protein partners relevant to the biological role of the protein.

No relatives of this two layer α+β fold can be discerned within current structure databases. Some topological relationship is detected to the Ivy virulence factor proteins (e.g. 2.5 Å rmsd over 71 residues of the *E.coli* Ivy protein (PDB 1GPQ [51]). Despite containing a five-stranded β-sheet and central helical components promoting a dimer interface, however, the many structural differences and lack of conserved sequence elements indicate the two families are not functionally related.

As well as possessing a novel fold, only two sequence homologs of **Vch_cass3** are known to date. A strain of *Desulfatibacillus alkenivorans* from polluted water (GenBank: CP001322) encodes a hypothetical protein sharing 37% identity. A second homolog (40% identity over available sequence) is present within a metagenomic sample of Antarctic marine bacteria. Sequence alignment shows strongest conservation of structural residues (e.g. Pro residues, C-terminal aromatic side chains) which are key to the dimeric α+β fold outlined.

In **Vch_cass3**, a long pronounced cleft (∼15 Å×19 Å) is located between the two sheets of each dimer, flanked by a number of acidic side chains (Figure 6C). This cleft is particularly Asp/Glu-rich, involving the segment of residues along the edge strand of the beta sheet, β5, and into helix 1. These exposed acidic residues at the open cleft are, however, entirely absent in the known sequence homologs of **Vch_cass3**. Thus, should this cleft form a functional binding site for the protein, its features would be unique to the biochemistry of the *Vibrio* gene cassette sequence alone.

#### Vch_cass14

**Figure 7:**
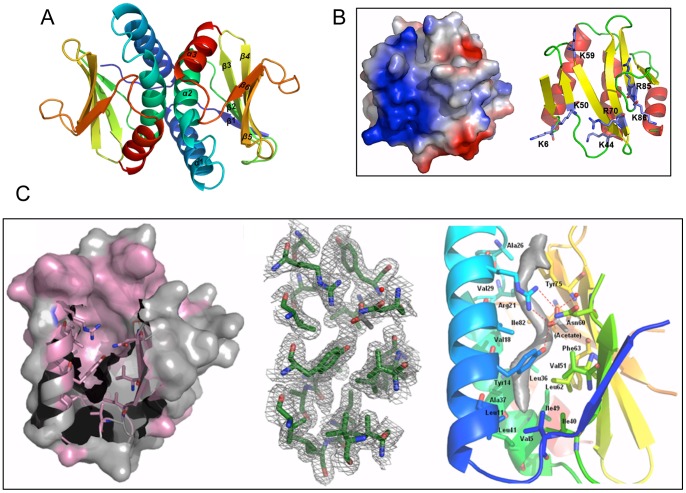
Vch_cass14 dimer with sequestered ligand (PDB 3IMO). **A.** Ribbon depiction of dimer coloured in spectrum from N-terminus (blue) to C-terminus (red) for each chain. **B.** Pronounced basic feature (in blue) dominates electrostatic surface of **Vch_cass14** (left); corresponding orientation of protein chain (right) outlines location of contributing Arg and Lys residues. **C.** Surface with ribbon representation (left) of the **Vch_cass14** binding pocket. Regions with ≥80% sequence conservation across the small family of homologs are coloured pink. Side chains contributing to the hydrophobic pocket are depicted. The 1.8 Å 2*F_o_-F_c_* map (contoured at 1σ level, mid-panel) shows side chains that line the extended cavity, as well as an area of electron density attributed to a sequestered small ligand. Residues lining this hydrophobic binding pocket are detailed (right panel). The single water molecule (red sphere) and unidentifiable ligand (grey) are also shown. The hydrogen-bonding network engaging Arg21, Tyr14, acetate and water is dashed in red.

The structure of **Vch_cass14** (PDB 3IMO, 1.8 Å) defines a small family of proteins of unknown function, other members of which occur in the genomes of soil-and water-dwelling bacteria (e.g. *Sorangium cellulosum*, sce0458; *Rhodopseudomonas palustris*, RPE_5052). **Vch_cass14** forms a dimer (an organisation confirmed in solution by size exclusion) in which each subunit adopts a two-layer α+β sandwich-type fold, as shown in Figure 7. Each chain forms a single anti-parallel sheet of six strands overlaid by a second face of three helices at a 45° angle. The topology order is relatively novel for this fold class: β1-α1-α2-β2-β3-β4-β5-β6-α3. The two protomers of the dimer interact orthogonally via their helix faces, with each central helix α2 making extensive hydrophobic contact across to all three helices of the paired module. A notable feature of the **Vch_cass14** dimer is the highly positively-charged surface displayed across each exposed β-sheet.

Internal to each monomer lies a particularly deep binding pocket formed by helices α1 and α2 and residues from the central four strands of the β-sheet (Figure 7C). The pocket is extensively lined with hydrophobic side chains (Phe73; Val5, Val18, Val29 and Val51; Leu11, Leu36 and Leu62; Ile15, Ile40, Ile49 and Ile82; Ala22, Ala26, Ala33 and Ala37; Tyr75) and could accommodate a ligand up to ∼15 Å in length. In the crystal form we have isolated, electron density consistent with a linear organic molecule is observed in this site, as shown in Figure 7. At the entrance to the pocket, a distinct cluster of polar residues (Arg21, Asn60 and Tyr14) is observed, engaged in this structure in a hydrogen bonding network with acetate and water molecules.

Across the known sequence relatives of **Vch_cass14**, similarity is relatively strong (46–62% identity), with sequences relating to helix α1, helix α2 and the connecting loop particularly well conserved (Figure S2). Amongst the three closest homologs (**Vch_cass14**, sce0458 and RPE_4052), all 20 of the internal pocket residues are invariant or conservatively substituted. The deep hydrophobic pocket observed in our **Vch_cass14** structure is thus likely retained as a binding site across this family of proteins. Mapping of other conserved residues onto the **Vch_cass14** structure additionally reveals a high degree of conservation for most hydrophobic side chains of the three helices, i.e. residues participating in dimer interactions. This most certainly points to a dimeric form being most relevant to biological function for this protein family.

## Discussion

The six new proteins whose structures are presented in this work were selected from integron gene cassette sequences on the basis of having no sequence similarity to any protein of known three-dimensional structure. The crystal structures we have since defined for these six proteins verify that the originating gene cassettes code for folded proteins which possess entirely new topologies. This attests to the notion that the integron/gene cassette metagenome is a source of a remarkable degree of biological diversity at the protein level, and that the genes encoded within it express functionally active proteins [52].

Although the structures of this set of integron/gene cassette proteins are novel, four of the six (**Vpc_cass2**, **Hfx_cass5**, **Vch_cass4** and **Vch_cass14**) share some similarity to small sequence groupings of undefined structure and function within gene databases. Thus, the information gained from the new structures featured here impacts beyond the specific structural targets to begin to delineate entirely new protein families and their associated members. In some examples, the identified sequence relatives of our set of integron-derived proteins are also themselves localised within gene cassettes.

For the majority, the structures described here provide features consistent with binding functions for the new proteins. The structure of **Vch_cass14** contains a deep cleft in which is sequestered an organic molecule incorporating an extended aliphatic chain plus acetate group. This is suggestive of a function consistent with an enzyme for catalysis, small substrate-sequestering protein or a transport protein. The structures of **Hfx_cass1** and **Hfx_cass2** each have surface clefts reminiscent of enzyme active sites. At present, the arrangements of the side chains in these clefts do not show exact template match to any previously defined active site chemistry, thus limiting any direct functional inference.

The **Hfx_cass5** family described here presents one of the most intriguing of the new protein structures. The structure is a tetramer built from two domain-swapped dimers. The tetramer is asymmetric, with one subunit from each domain-swapped dimer forming a central contact with its equivalent subunit from the other dimer. This contact is mediated by a 3_10_ helix whose sequence is strongly conserved in related proteins. On one face of the **Hfx_cass5** tetramer, the two closest domains of separate dimer groupings form a narrow slot that is lined with basic side chains. This creates the appearance of a binding site for an extended, acidic ligand. Because of the asymmetry of chain packing, the equivalent (far wider) slot on the opposite face of the oligomer lacks such coherent binding-site features. It is tempting to speculate this protein could act as a bistable switch, binding an elongated acidic ligand first on one face and then the opposite, dependent upon the disposition of the (conserved) 3_10_-helices.

Although not correlating to known binding site geometries, the six proteins in this study display features consistent with putative binding sites, either for interaction with small molecule ligands or, potentially, other protein partners. All six are relatively small (<22 kDa) proteins and, notably, each engages in multimerisation to form larger complexes in solution. A tendency to form homo-oligomers in solution and/or crystal has been a consistent observation across our structural survey of gene cassette proteins, including our earlier structures of the dimeric proteins Bal32 and iMazG [52], [53]. We have only observed one exception to date, a cationic drug-binding protein from *Vibrio* [54]. This clear preference for oligomerisation may be a consequence of the relatively short sequence lengths of genes cassettes within arrays, stabilising small protein modules which can perhaps also be readily and flexibly mixed for different functions. Such modules which in isolation form homo-oligomers, may in concert with appropriate catalytic, binding or membrane domains eventually become integrated within more sophisticated biochemical machinery or regulatory networks. Certainly, the surface features we have described for each of our cassette protein structures would have capacity to act as heterogeneous protein interfaces within multidomain or multiprotein systems. However, should the appropriate connected subunits themselves also be encoded on mobile gene cassettes, there may be limitations to the rapid evolution of protein complexes that are fully functionally fit or capable of providing niche advantage.

In the case of **Vpc_cass2**, our structural approach has identified some distant homology with the C-terminal domain of kanamycin nucleotidyltransferase. Like its remote structural homologs, **Vpc_cass2** forms a homodimer, although in this case through a distinctively new stacking geometry. Thus, the shape of the dimeric interface differs to that found in the relative KNTase-C, HI0074 from *Haemophilus influenza*. In HI0074, the overall dimer is distinctly curved and somewhat resembles a DNA-binding surface, although no such activity can be experimentally observed [55]. An adjacent gene on the *H. influenzae* genome encodes the protein HI0073 which is known to associate with HI0074 in a stable complex, also possibly at this interface [55]. For **Vpc_cass2**, the exposed surfaces of the dimer are relatively flat, with a distinct conserved cluster of charged residues straddled across the interface, so more suggestive of a protein (rather than any DNA) interaction site.

At the genetic level, genes for bacterial NTase substrate-binding proteins are often found adjacent to separate nucleotidyltransferase genes, i.e. those encoding the corresponding catalytic subunit. In the case of KNTase, the two relevant elements are actually encoded within a single gene, so containing both the helical substrate-binding domain and the distinct α/β nucleotide-binding domain positioned about a long binding-site cleft [56]. Lehmann et al. have documented substrate-binding/nucleotide-binding module pairs to be quite prevalent in bacterial genomes, particularly from harsh conditions and pathogens [55]. Thus, the mobile gene cassette element **Vpc_cass2** could serve as an example of one half of a bipartite system, with the capacity to become established with a nucleotidyltransferase second domain to provide a functional enzyme offering selective advantage.

Our structural study has shown that the highly novel (∼80% unknown) gene cassette metagenome is not merely a repository of sequence divergent variants of known proteins, but in fact mobilises a repertoire of genes belonging to new, currently uncharacterised protein families. The significant structural spread we observe across these six representatives of cassette-proteins suggests each to either perform a biological function hitherto undescribed in other bacterial proteins, or to achieve a known function by a new mechanism, and thus apt to be under different regulatory control. In either scenario, it is probable that the phenotypes provided by cassette proteins expand the functional repertoire of the recipient organism, just as might also be provided by other types of mobilised features within, for instance, genomic islands. This hypothesis is strengthened by the persistence of sequence homologs in the cassette metagenome across widely varying geographical locations experiencing similar environmental stressors. The high degree of novelty that we consistently observe across sequences and structures of integron/gene cassette proteins attests to the fact that this pool of genes remains relatively uncharacterised by normal sequencing efforts. Thus, to fully characterise and understand the global proteome, it remains essential to continue to independently target the metagenomic element.

## Supporting Information

Figure S1 **Sequence alignment of Hfx_cass5 and related cassette-proteins.** Secondary structure elements are from **Hfx_cass5**, as determined in this work. Invariant (white characters, black shading) and chemically equivalent (black characters, grey shading) residues across ≥80% of the family are shown. Red dots delineate exposed residues engaged in tetrameric interaction. Sequences are: (**Hfx_cass5**) cassette protein sourced from a raw sewage effluent outfall in the North West Arm, Halifax, Canada; (B0BGV4) cassette protein sourced from the same sewage outfall; (B0BK21) cassette protein sourced from a geographically distinct raw sewage effluent outfall in Halifax Harbour, Halifax, Canada; (Bal50) cassette protein sourced from soil contaminated with industrial waste, at an electricity power station in Balmain, Sydney, Australia. (Pxanth) *Pseudoxanthomonas suwonensis* 11-1 from compost-feedstock enrichment culture, bioreactor, USA [58]; (Shew2602) *Shewanella loihica* PV-4 from deep sea hydrothermal vent near Hawaii, Pacific Ocean [59].
(DOCX)

Figure S2 **Sequence alignment of Vch_cass14 and its related sequences.** Secondary structure elements are from **Vch_cass14**, as determined in this work. White characters with black shading are indicative of identical residues across the three sequences. Black residues on grey shading indicate chemically equivalent residues shared by all sequences. Red dots and blue triangles delineate residues forming pocket surface and dimer interface, respectively. Sequences are: (**Vch_cass14**) cassette isolated from the chromosomal integron of *V. cholera*; (sce0458) hypothetical protein encoded by *S. cellulosum*; (BosA53) hypothetical protein encoded by *R. palustris*.
(DOCX)
