# Supplementary figures and images for: Integron Gene Cassettes: A Repository of Novel Protein Folds with Distinct Interaction Sites
Source: PLoS One. 2013 Jan 18;8(1):e52934. doi: 10.1371/journal.pone.0052934 (PMC3548836; doi:10.1371/journal.pone.0052934)

**Figure S1**


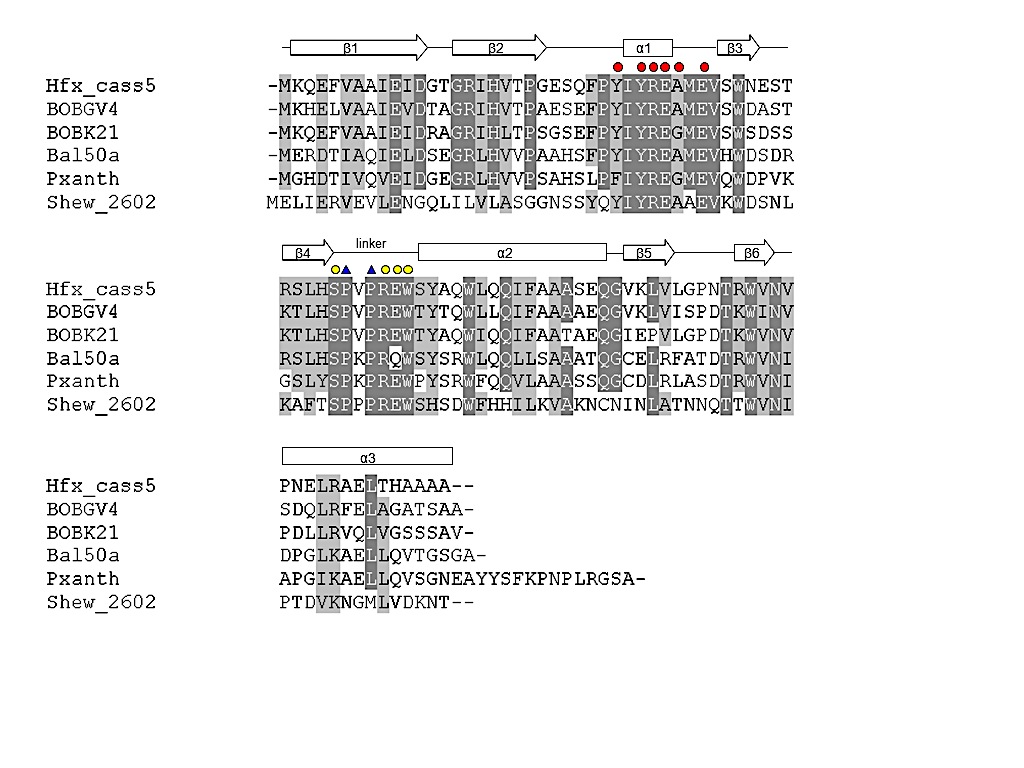

Supplement: Figure S1 — Sequence alignment of Hfx_cass5 and related cassette-proteins. Secondary structure elements are from Hfx_cass5, as determined in this work. Invariant (white characters, black shading) and chemically equivalent (black characters, grey shading) residues across ≥80% of the family are shown. Red dots delineate exposed residues engaged in tetrameric interaction. Sequences are: (Hfx_cass5) cassette protein sourced from a raw sewage effluent outfall in the North West Arm, Halifax, Canada; (B0BGV4) cassette protein sourced from the same sewage outfall; (B0BK21) cassette protein sourced from a geographically distinct raw sewage effluent outfall in Halifax Harbour, Halifax, Canada; (Bal50) cassette protein sourced from soil contaminated with industrial waste, at an electricity power station in Balmain, Sydney, Australia. (Pxanth) Pseudoxanthomonas suwonensis 11-1 from compost-feedstock enrichment culture, bioreactor, USA [58]; (Shew2602) Shewanella loihica PV-4 from deep sea hydrothermal vent near Hawaii, Pacific Ocean [59]. (DOCX) [file pone.0052934.s001.docx]

**Figure S2**


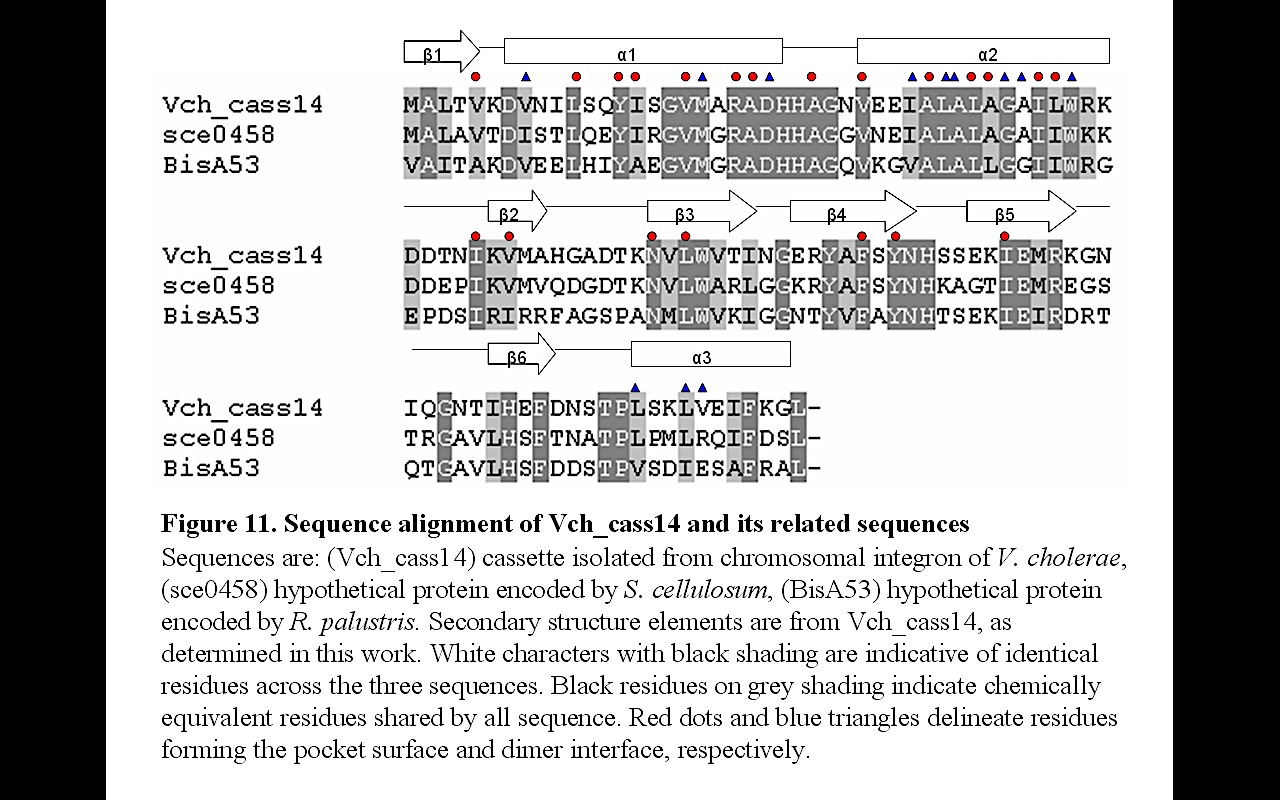

Supplement: Figure S2 — Sequence alignment of Vch_cass14 and its related sequences. Secondary structure elements are from Vch_cass14, as determined in this work. White characters with black shading are indicative of identical residues across the three sequences. Black residues on grey shading indicate chemically equivalent residues shared by all sequences. Red dots and blue triangles delineate residues forming pocket surface and dimer interface, respectively. Sequences are: (Vch_cass14) cassette isolated from the chromosomal integron of V. cholera; (sce0458) hypothetical protein encoded by S. cellulosum; (BosA53) hypothetical protein encoded by R. palustris. (DOCX) [file pone.0052934.s002.docx]
